# Comprehensive Analysis of a *Vibrio parahaemolyticus* Strain Extracellular Serine Protease VpSP37

**DOI:** 10.1371/journal.pone.0126349

**Published:** 2015-07-10

**Authors:** Monica Salamone, Aldo Nicosia, Carmelo Bennici, Paola Quatrini, Valentina Catania, Salvatore Mazzola, Giulio Ghersi, Angela Cuttitta

**Affiliations:** 1 Laboratory of Molecular Ecology and Biotechnology, National Research Council, Institute for Marine and Coastal Environment (IAMC-CNR), Detached Unit of Capo Granitola, Torretta Granitola 91021, Trapani, Sicily, Italy; 2 Dipartimento di Scienze e Tecnologie Biologiche, Chimiche e Farmaceutiche (STEBICEF),Università di Palermo, Viale delle Scienze, edificio 16, Palermo, Sicily, Italy; 3 National Research Council, Institute for Marine and Coastal Environment (IAMC-CNR), Calata porta di Massa, 80133, Napoli, Italy; 4 ABIEL S.r.l., Via del Mare 3, Torretta Granitola 91021, Trapani, Sicily, Italy; Stanford University, UNITED STATES

## Abstract

Proteases play an important role in the field of tissue dissociation combined with regenerative medicine. During the years new sources of proteolytic enzymes have been studied including proteases from different marine organisms both eukaryotic and prokaryotic. Herein we have purified a secreted component of an isolate of *Vibrio parahaemolyticus*, with electrophoretic mobilities corresponding to 36 kDa, belonging to the serine proteases family. Sequencing of the N-terminus enabled the *in silico* identification of the whole primary structure consisting of 345 amino acid residues with a calculated molecular mass of 37.4 KDa. The purified enzyme, named VpSP37, contains a Serine protease domain between residues 35 and 276 and a canonical Trypsin/Chimotrypsin 3D structure. Functional assays were performed to evaluate protease activity of purified enzyme. Additionally the performance of VpSP37 was evaluated in tissue dissociations experiments and the use of such enzyme as a component of enzyme blend for tissue dissociation procedures is strongly recommended.

## Introduction

Proteases are a highly valuable enzymes, which can be classified into four main groups based on the essential catalytic residue at their active site: serine proteases, cysteine proteases, aspartate proteases and metalloproteases.

Widely expressed in eukaryotes, prokaryotes, archaea, and viruses, serine proteases exert several physiological activities, including digestion, haemostasis, apoptosis, reproduction and immune response. Moreover their sequential activation of drive blood coagulation, fibrinolysis and complement cascade are well studied [1, 2]. Similar mechanisms are involved in the process of embryo development, matrix remodelling, differentiation, and wound healing [3]. The first mechanism of action of serine peptidases was discovered by the kinetic studies of chymotrypsin by Bender and his co-workers[4]. For a long time only two groups of serine peptidases were known; the trypsin and subtilisin groups. With the development of molecular biology procedures and the determination of three-dimensional structures, a variety of other serine peptidases were discovered [5].

To date, several applications of proteolytic enzymes have been reported in the field of regenerative medicine, including tissue dissociation for tissue engineering, such as the isolation of pancreatic Islet for transplantation procedures [6–8]. Tissue disgregation is usually performed at 37°C in presence of collagenases and proteases such as Neutral protease from *Clostridium histolyticum* or Thermolisin from *Bacillus thermoproteolyticus rocco*. These enzymes are able to activate pancreatic proenzymes, which show optimum activity at the same temperature. Therefore a decrease of islets yield through fragmentation and disintegration has been reported [8].

So that, a challenge in tissue dissociation procedures is the identification of proteases working at a lower temperature than 37°C.

To provide a variety of proteases, new sources of proteolytic enzymes, including proteases from fish [9, 10] and from marine bacteria [11] have been studied and identified.

It has been shown that marine enzymes have unique properties including the high efficiency at lower temperatures and the ability to be inactivate with a simple raising of temperature [12]. Additionally, since enzymes from marine microorganism are known to be more stable and active than animals’ and plants’ enzymes, large interest on these microbial sources of enzymes has arisen [13,14].

The growing demand of these kinds of enzymes has led to clone and express proteases as trypsin in heterologous expression systems [15, 16].

Several members of Trypsin and Chymotrypsin families are produced as inactive precursors and secreted in the extracellular milieu. Even if such family mainly consists of eukaryotic enzymes, it has been shown that proteases related to trypsin are usually produced by *Streptomyces omiyaensis* and *Streptomyces griseus* [16–18]. Additionally, three different proteases, named as VesA, VesB and VesC, structurally related to trypsin, have been identified as components of secretome of *Vibrio colerae* via the Type II Secretion mechanism [19, 20]. Also bacteria belonging to *Vibrio parahaemolyticus* are known to produce several proteases and collagenases including metalloproteases of the zincins superfamily and serine proteases [21–24]. Since the sequencing of clinical isolate RIMD2210633 was published [25], a lot of draft genome sequences for *V*. *parahaemolyticus* strains have been produced [26] therefore a large dataset of *V*. *parahaemolyticus* nucleotide sequences is now publicly available. Since development of digital genomic/transcriptomic platforms has allowed the discoveries of novel members of known classes of proteins as well as novel factors previously unknown in different organisms [27, 28], the analysis of public available databases can facilitate proteomic exploration [29, 30]. Marine bacteria such as *Vibrio* spp., *Shewanella putrefaciens*, and *Staphylococcus* and *Micrococcus* spp are known to cause wound infections in humans [31, 32]. Fish bites are rare causes of these wounds [33] and shark species have been shown as being primarily involved [34]. Moray eels, generally regarded as aggressive, are able to attack humans and their bites represent a potential cause of serious bacterial infections [35–39].

Herein microbiological and biochemical procedures were combined with *in silico* analysis and homology modeling studies to identify a new protease belonging to the serine proteases family and herein named as VpSP37. The identified enzyme showed half of maximal activity at 25°C; therefore its use in islet isolation techniques can overcome problems associated to inappropriate pancreatic enzyme activation as islet fragmentation and isolation failure.

## Results and Discussion

### Isolation of bacterial strain

**Fig 1:**
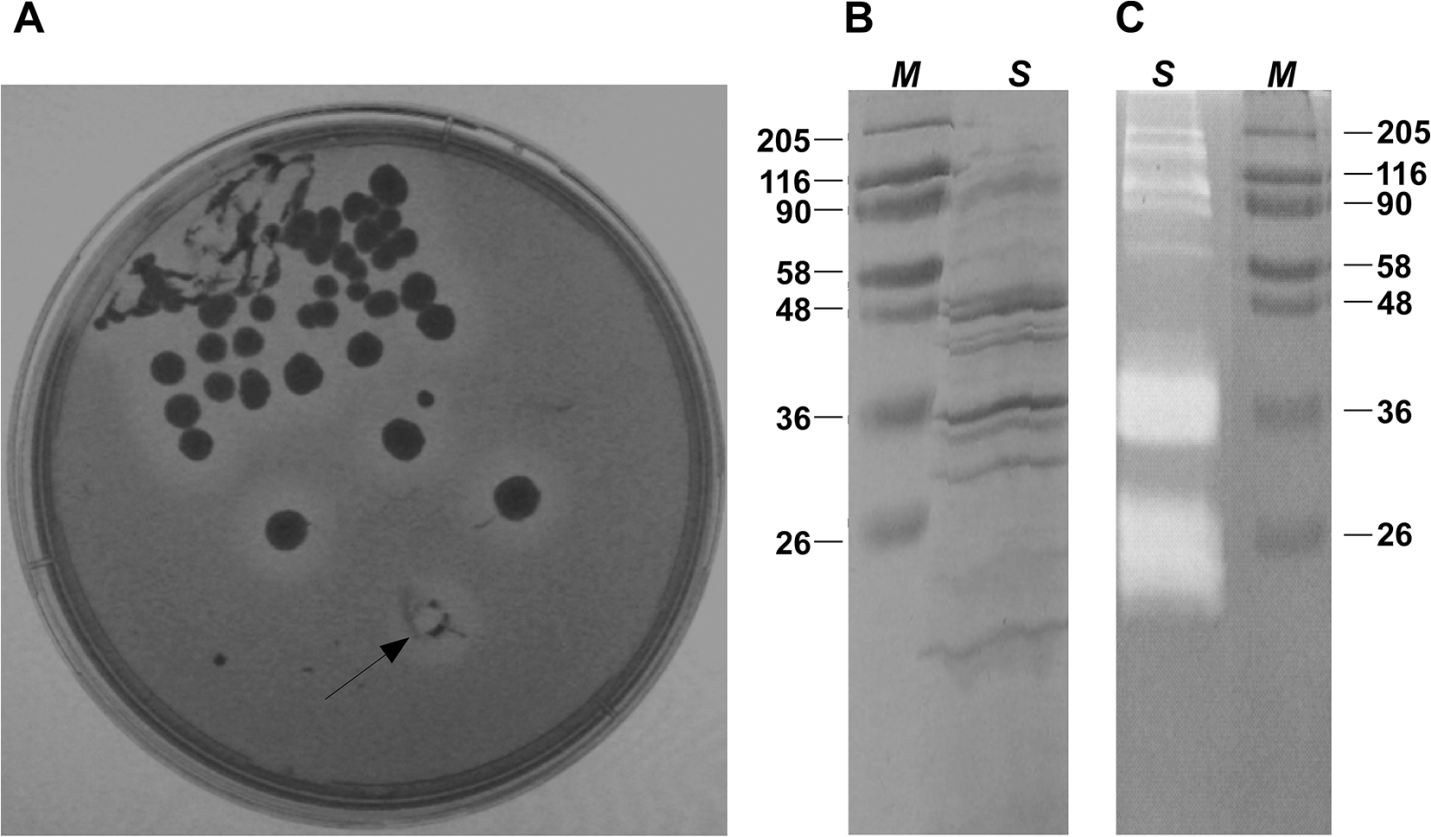
Detection of extracellular gelatinolitic degradation by gelatin-agar plate and zymography. (A) gelatin agar plates were streaked with the *Vibrio* isolate B2 from the oral cavity of *M*. *helena* for 24 Hrs. Agar plate was then stained with Red Ponceau to identify the degradation of gelatine by extracellular secreted enzyme. The arrow indicates the colony used for subsequent studies. (B-C) SDS-PAGE and zymography respectively of isolate B2 secreted proteins. The supernatants obtained from cultures were filtered, precipitated and 10 μg (B) or 1 μg (C) were loaded (Lane S). Lane M indicates molecular weight marker proteins (Prestained Molecular Weight Marker, Sigma). Samples were loaded on a 10% acrylamide gel.

In order to provide novel enzyme sources, a *Vibrio* strain (isolate B2) was recovered from the oral cavity of the Mediterranean eel *Muraena helena*. Microscope observations (data not shown) revealed that the isolate is a motile Gram negative rod. The RDP Classifier analysis assigned the 1150 nt sequence of its 16S rRNA gene to the genus *Vibrio* (100%). The BLAST search performed using the EMBL/SwissProt/GenBank non-redundant nucleotide database showed that the closest related sequence belongs to an uncultured *Vibrio* sp (clone HA_42, 99% id, Score 2080; Zhou,G unpublished). BLASTing the sequence against the restricted 16S rRNA gene database the closest relative is *V*. *parahaemolyticus* strain NBRC 12711 with an identity of 99% (Score: 2063) followed by *V*. *alginolyticus* with 99% identity and a slightly lower score (Score: 2047). *Vibrio* are known to produce several extracellular proteolytic enzymes to modulate the bacterial virulence [40]. Thus, in order to evaluate the production of secreted proteases, the isolated strain of *V*. *parahaemolyticus* was streaked on agar plate containing 2% gelatine (Fig 1A). After 24 hrs, all analysed colonies showed a ring of gelatine degradation which confirmed the production of extracellular proteases with gelatinolytic substrate activity.

### Purification and characterization of extracellular proteolytic enzymes

**Fig 2:**
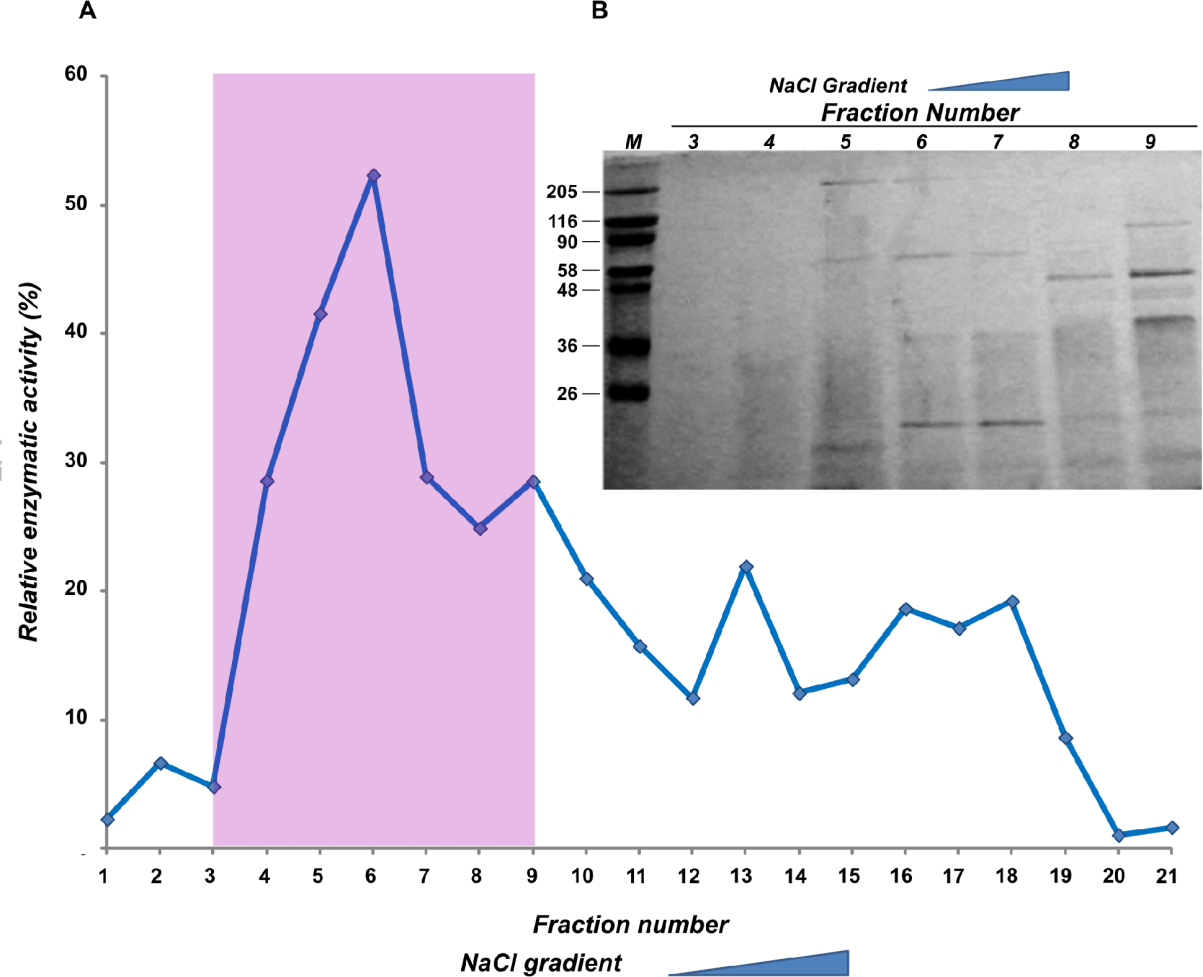
Enzymatic efficiency of fractions from the *Vibrio isolate* supernatants. (A) Proteins were separated using anionic exchange chromatography and to investigate proteinase activity, casein was used as substrate. (B) The fractions from 3 to 9 showing higher activity were analysed by SDS-PAGE.

**Fig 3:**
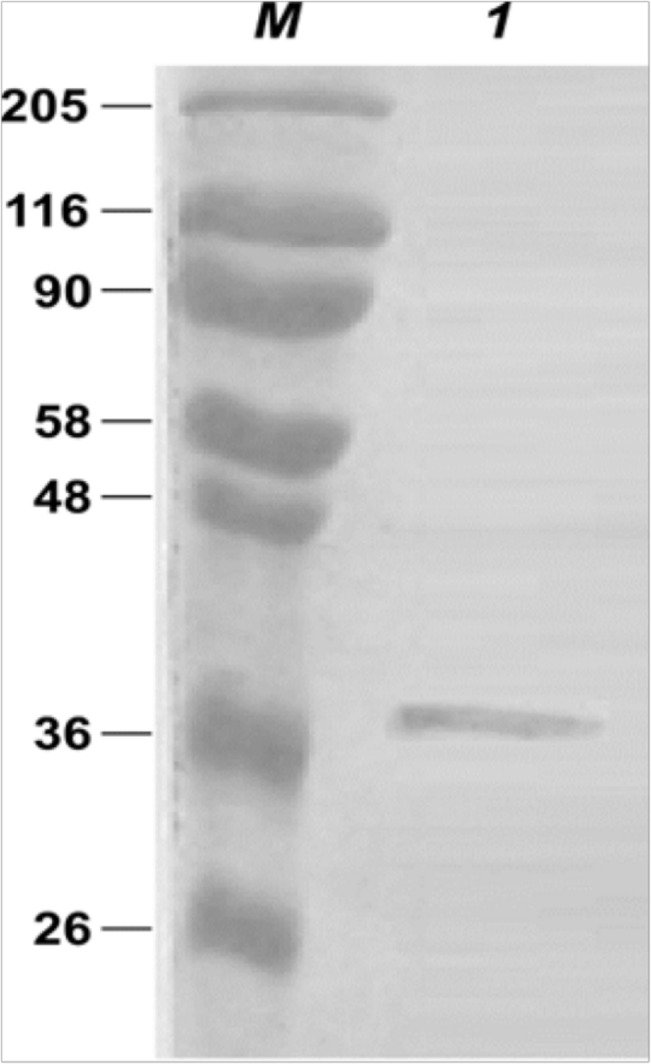
SDS-PAGE of purified protease from *Vibrio isolate B2*. Lane M indicates molecular weight marker proteins (Prestained Molecular Weight Marker, Sigma). In lane 1 2μg of purified protein were loaded. Samples were loaded on a 10% acrylamide gel.

In order to evaluate the complexity of extracellular milieu secreted proteins and to study the proteolytic activities released from bacterial cells, supernatants obtained from cultures of *V*. *parahaemolyticus* strain B2 were filtered, precipitated, and subjected to SDS PAGE and zimography analyses (Fig 1B). These analyses retrieved a discrete pattern of bands, suggesting the presence of several proteases and proteins complexes. In particular a band of 90 kDa were found to have gelatinolytic activity, which was inhibited by EDTA (data not shown). This gelatinase probably corresponds to the metalloprotease previously identified as VppC, belonging to the zincins super family [21]. Moreover, two major components showing gelatinase acitivity, with a molecular weight of about 36 KDa and 25 KDa respectively, were identified. Up to this day, no study has provided data focused on these gelatinases in *V*. *parahaemolyticus* In order to investigate the proteolytic efficiency of these two components, they were incubated with insoluble collagens or casein and their activities were compared with recombinant *C*. *histolyticum* collagenases together with Neutral Proteases (commercially available), currently used for tissue dissociation procedures. As shown in Table 1, the supernatants of strain B2 contain both collagenolytic and caseinolytic activities, likely suggesting to contain highly active proteases; obviously, commercial proteases and collagenases activities are the highest; it is due to a high degree of purity. Moreover, collagenases from *C*. *histolyticum* are reported to be more active on insoluble collagens compared to many other collagenases [41]. In order to fractionate secreted proteases, the crude proteolytic mixture was then subjected to anion exchange chromatography and the enzymatic activity of the proteases present on different fractions were investigated using casein as substrate. As shown in Fig 2A, major activity was found in fraction number 6. Therefore, we deduced that in this fraction highly active proteases were present. Because such fraction is composed of at least of 4 different protein species (Fig 2B), a molecular size exclusion chromatography was required for their separation and identification. Proteins of fraction number 6 were resolved by gel filtration and analysed for their caseinolytic activity (data not shown). The fraction ranging from 30 to 40 KDa displayed the highest activity and as shown in Fig 3 it consists of a single protein with a molecular weight, presumably corresponding to 37 KDa.

**Table 1:** Comparison of collagenolytic and caseinolytic activities of *V parahaemolyticus* secreted proteases and enzymes commonly used for tissue dissociation.

	Collagenolytic activity(U/mg)	Caseinolytic Activity(U/mg)
Supernatants *V.parahaemolyticus*	74.6 ±2	118.6±1
Collagenases *C. histolyticum*	900±3	n.d.
Neutral Protease *C. histolyticum*	n.d.	829.9±5

In the enzymatic test, one unit liberates peptides from collagen or casein equivalent in ninhydrin color to 1.0 μmole of leucine in 5 hours at pH 7.4 at 37°C in the presence of calcium ions.

### Identification of protease

The purified protein, separated by SDS–PAGE under reducing conditions, was transferred to PVDF membrane and sequenced. The N-terminal sequence was determined by automated Edman degradation. As a result, an 18-mer peptide (SDGSELDEQGISTAIIGG) was obtained and used as a query for Blast researches against public available sequences database. This analysis revealed a high similarity with Chymotrypsin family members, whereas 100% identity was obtained with several identical serine proteases which have been computationally annotated from the *V*. *parahaemolyticus* genome.

The protein consists of 345 amino acid residues with a calculated molecular mass of 37378.4 Da and contains a Trypsin-like serine protease domain between residues 35 and 276. The protein is synthesised as inactive precursors with a putative tripartite N-terminal signal peptide (residues 1–21), required for translocation across the inner membrane via the Sec pathway, consisting of a positively charged N-terminal region (n-region, residues 1–6), a hydrophobic central region (h-region, residues 7–15) and a neutral, polar C-terminal region (c-region 16–21). As an important feature of chymotrypsin family of serine proteases, a cleavage site for proteolytic activation located between residues Ala21 and Ser22 was predicted by the SignalP algorithm. These results were consistent with the above discussed sequencing by Edman degradation and indicated that the protease, similarly to the members of trypsin like family, has been cleaved at Ala21—Ser22.

**Fig 4:**
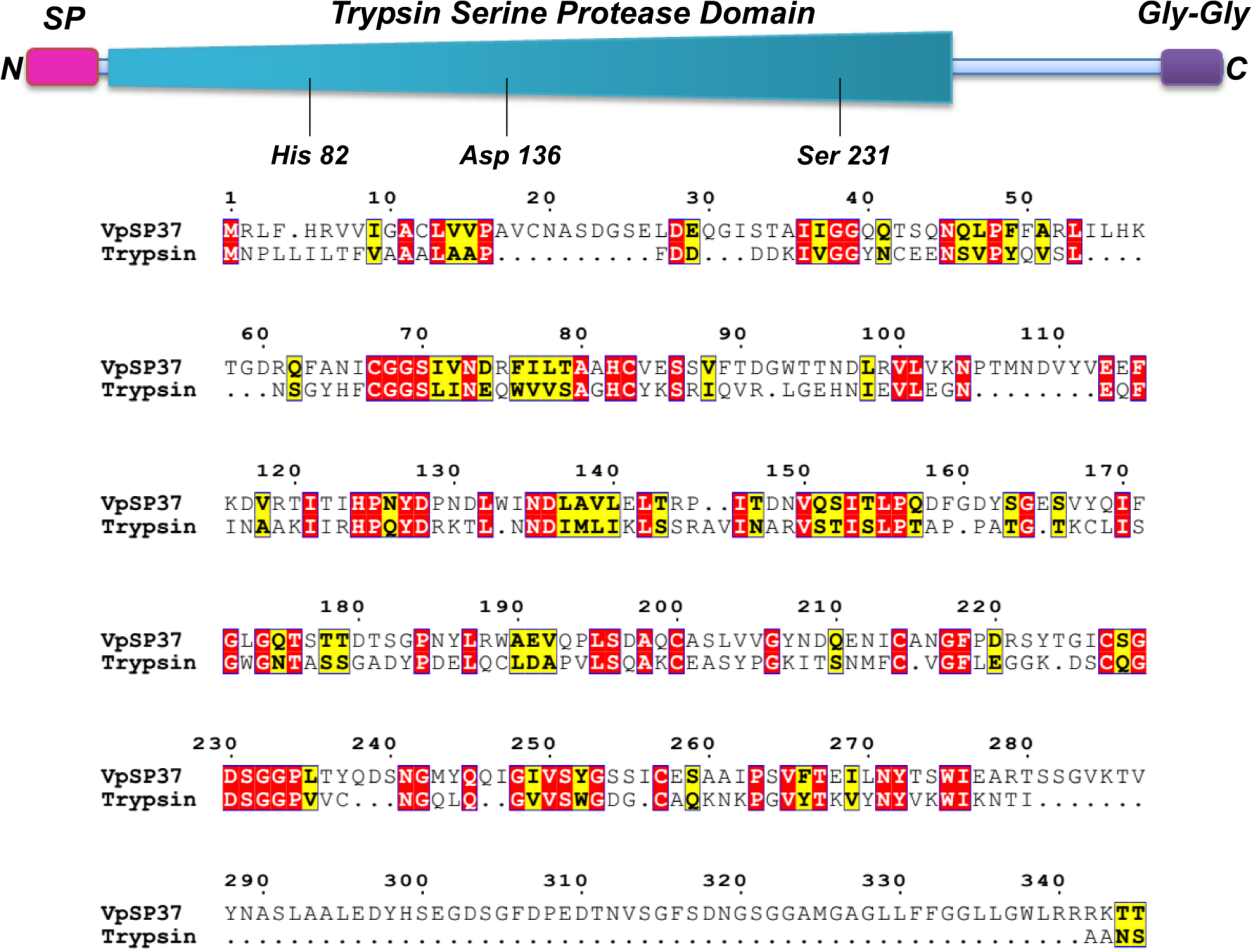
Schematic diagram of VpSP37 and identification by protein alignment of catalytic triad comprised of His Asp and Ser. (On the top) Protease possesses a N-terminal signal peptide in pink, a protease domain in pale blue and a C-terminal Gly-Gly repeat in purple. Amino acids forming the catalytic triad are shown. (On the bottom) Sequence alignment of VpSP37 and human trypsinogen. Similar residues are written in black bold characters and boxed in yellow, whereas conserved residues are in white bold characters and boxed in red. The alignment was performed with T-coffe. The sequence numbering on the top refers to the alignment.

These findings enabled us to consider this protein to be representative of the bacterial Serine proteases family and we designated it as VpSP37(for *V* *ibrio* *p* *arahaemolyticus* secreted protease 37 kDa) (Fig 4).

Comparative homology studies between the protease domains of VpSP37 and Trypsinogen revealed that the protease both the proteases possess the catalytic triad characteristic for serine proteases, consisting in the conserved His82, Asp136, and Ser231 residues, and an arrangement of amino acids which clearly falls into the S1 A chymotrypsin family.

**Fig 5:**
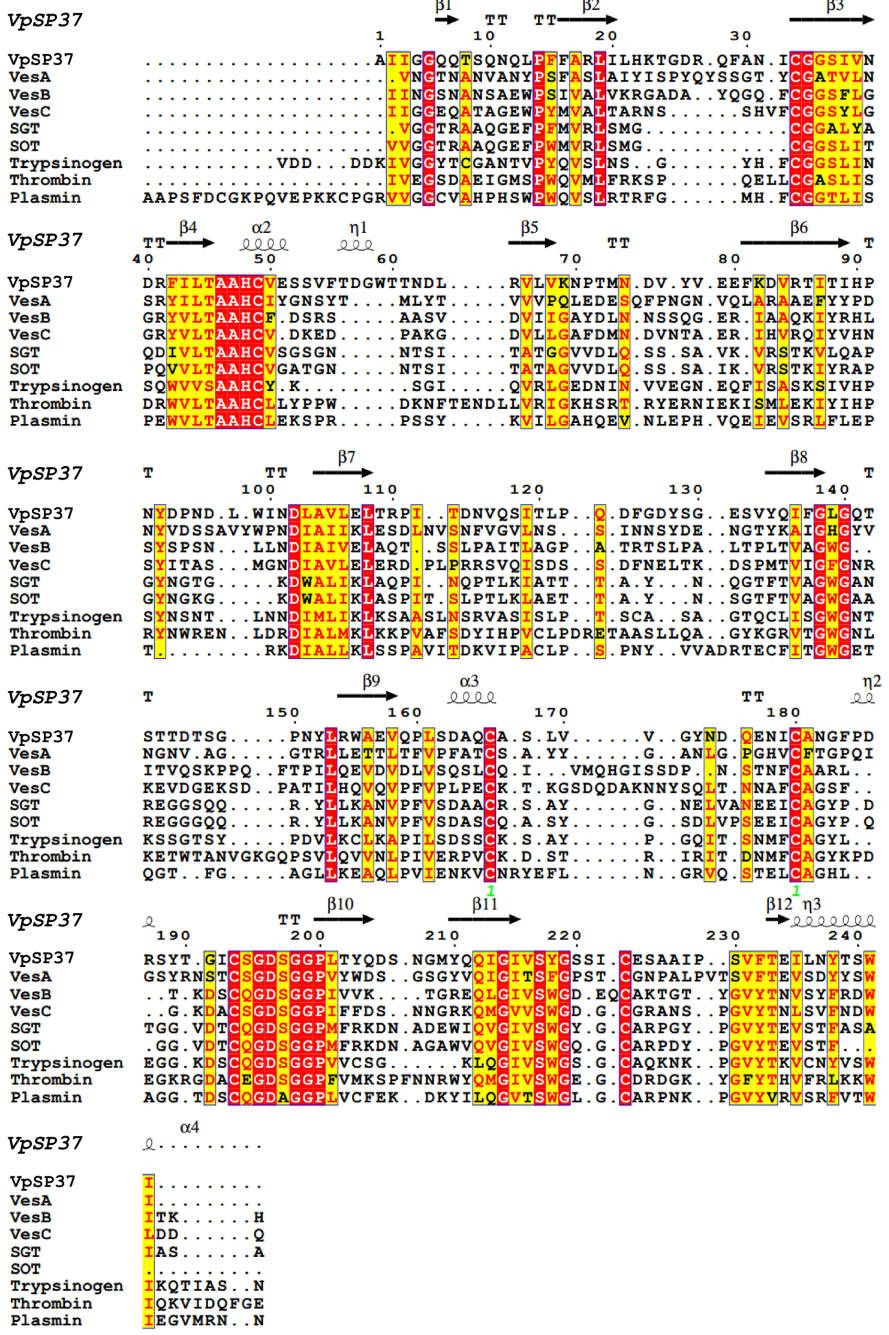
Multiple sequence alignment of mature *VpSP37* with other trypsin like serine proteases. Alignment was performed with T-coffe. Secondary structure elements of VpSP37 are shown above the sequences block, similar residues are written in black bold characters and boxed in yellow whereas conserved residues are in white bold characters and boxed in red. The sequence numbering on the top refers to the alignment. Abbreviations, species, and accession numbers are listed in Table 2.

Even if this family mainly consists of eukaryotic proteases, a few serine proteases have also been identified in *S*. *omiyaensis*, *S*. *griseus* and *V*. *colerae*. Thus Blast analysis showed that homologous proteins are widely distributed among Vibrionacee members but poorly represented among Gram-negative bacteria. Therefore a multiple sequence alignment was also constructed for the mature form of VpSP37 and bacterial/eukaryotic proteases (see Table 2 for details) in order to identify conserved structures or motifs (Fig 5). Mature VpSP37displayed identity ranging from 29% to 32% with Ves proteins from *V*. *cholerae*, and SGT and SOT proteases from *S*. *omiyaensis* and *S griseus*. Noteworthy similar results (26% to 31% identity) were also obtained with eukaryotic serine proteases as Trypsinogen, Thrombin and Plasmin.

**Table 2:** Trypsin like serine proteases used in this study.

Species	Protease	GenBank accession number
*Homo sapiens*	Prothrombin	AAC63054.1
	Plasminogen	NP_000292.1
	Trypsinogen	AAA61232.1
*Bos taurus*	Trypsinogen	NP_001107199.1
*Streptomyces omiyaensis*	SOT	AB362837
*Streptomyces griseus*	SGT	P00775
*Vibrio cholerae*	Ves A	VCA08039
	Ves B	VC1200
	Ves C	VC1649

An additional hall mark feature of trypsin-like serine protease is an Aspartate residue which is located at the bottom of the S1 pocket (in the position 189 according to the chymotrypsin numbering). It has been considered a primary determinant of arginine and lysine specificity attracting and stabilizing a positively charged arginine or lysine residue in the substrate [42]. Conversely, enzymes exhibiting elastase specificity, lack Asp 189 and show a hydrophobic depression which provides a platform for interaction with small P1 substrate side chains.

In VpSP37 this residue is a Gly (Gly225 in Fig 4) which would not be able to engage in the obligatory salt bridge interaction with the P1-Arg guanidinium group. Hence it could be hypothesized that VpSP37 may likely bear elastase-like specificity determining residues in the active site

### Homology Modelling Analysis

Amino acid sequences diverge more rapidly in evolution than the 3D-structure, therefore to evaluate the substrate specificities and to assess the presence of conserved structural motifs, the 3D structure of the VpSP37 was predicted by homology modelling using 6 templates (PDB id: 4lk4A_; 4durA_; 2b9lA_; 2f83A_; 4hzhB_ 3nxpA_) selected to model VpSP37 based on heuristics to maximise confidence, percentage identity and alignment coverage. The structures corresponding to N- and C-termini (86 residues) were modelled by *ab initio* and the generated model was validated by assessing a Ramachandran plot. The results of these analyses showed that the percentage of residues in the favoured/allowed region ranged from 90% to 95%.

**Fig 6:**
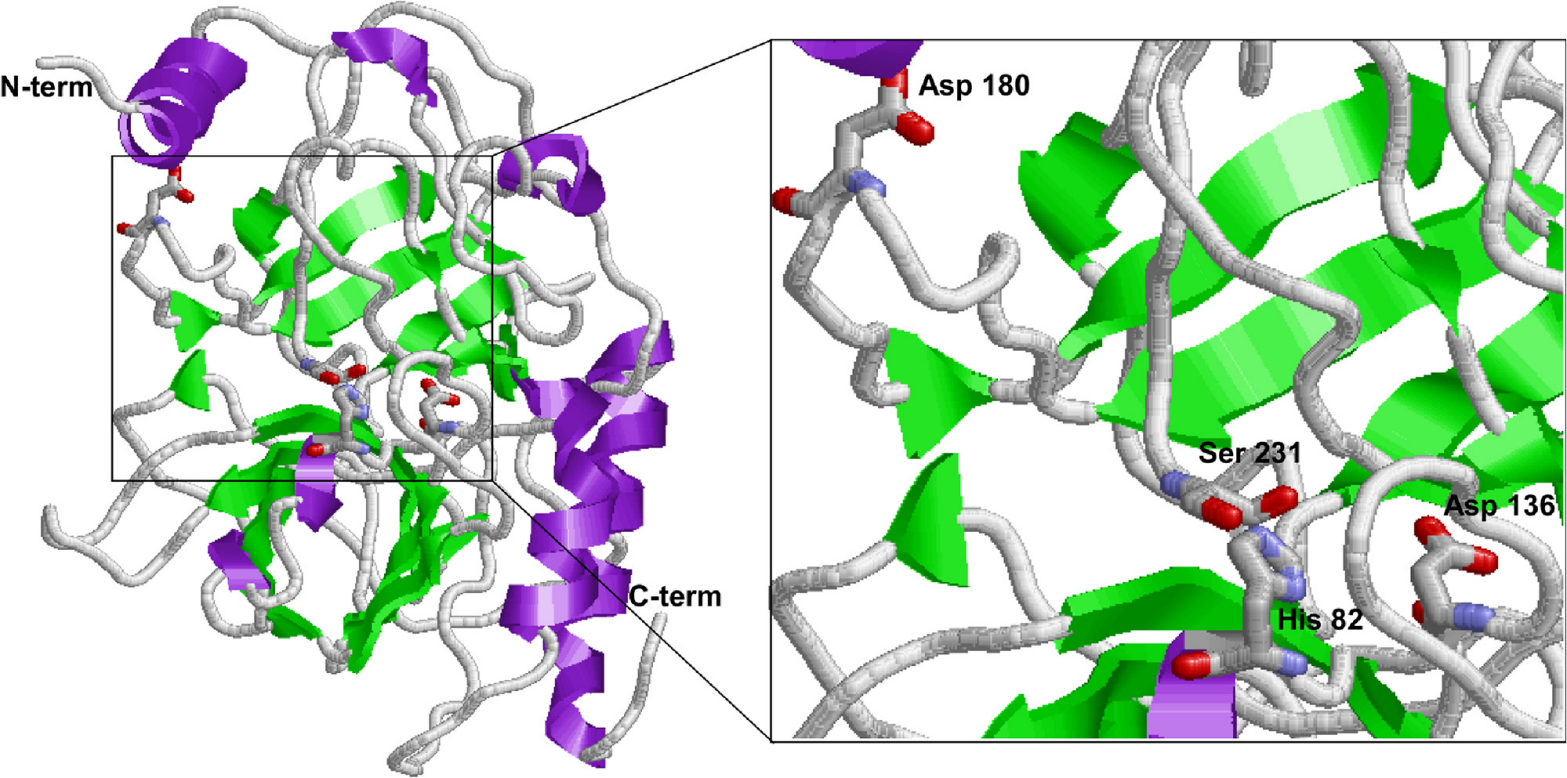
Homology modelling of VpSP37. The 3D model structure is shown in ribbon representation with α-helices in purple and β-strands in green. The catalytic triad residues His 82, Asp 136 and Ser 231 are shown.VpSP37 3D structures were reconstructed by homology modelling. The 3D structures of six different templates (4lk4A_; 4durA_; 2b9lA_; 2f83A_; 4hzhB_ 3nxpA) were used as template.

Because VpSP37 was modelled in an inactive form, residues 1–21, corresponding to the N-terminal signal peptide is showed as α helices; additionally the model predicted the presence of loops and β strands organised in super secondary structures. VpSP37 possesses a fold consisting of two β-barrel domains with similar topology (Fig 6) and composed of 6 antiparallel β-strands. The catalytic triad, previously identified, is located at the interface of the two domains. According to comparative analysis, the S1 pocket wall is formed by by three β-strands (β-10, 11 and 12 in Fig 5) connected by the disulphide bond Cys227 and Cys257 at the C-terminal β-barrel domain. Such structure corresponds to the typical trypsin/chymotrypsin fold [43] in which amino acid side chains, which form the S1, are responsible for the specificity. Disordered regions located nearby the active site were also predicted and may be reasonably considered as a reminiscence of the disordered loops already described in the bovine trypsinogen structure [44].

Despite the absence of an Asp residue at position 225 inVpSP37 it is possible to identify at least four different Asp amino acid residues located nearby the S1 pocket (Asp180-197-209-220). VpSP37 residues 197, 209 and 220 seem to be located away from the top of the S1 pocket. Conversely, Asp180 was found to be spatially conserved as it was predicted to adopt its position and orientation at the bottom of the S1 pocket, similar to those of trypsin family members. Hence it could be hypothesised that Asp180 may likely be engaged in the obligatory salt bridge interaction as occurred in trypsin.

During the years several data focused on S1 site specificity of enzymes with chymotrypsin folds have been produced. In this scenario, it has also been shown that Asp 189 substitution with other amino acids does not alter substrate specificity. Additionally, although the human neutrophil serine protease 4 (NSP4) has been shown to bear an elastase-like S1 pocket, it exhibits a trypsin-like specificity for cleaving substrates after arginine residues [45]. Therefore alternative mechanisms for substrate specificities in Trypsin-fold proteases are possible.

### Characterization of VpSP37 activity

From these considerations it becomes clear that a refinement of S1 site specificity in VpSP37 required the characterization of enzyme activity.

To functionally assess the enzymatic activity of VpSP37 and to confirm the trypsin-like substrate specificities, the purified protein was separately incubated in the presence of specific synthetic peptides for the evaluation of trypsin, elastase like and chymotripsin activity. The results are shown in Table 3.

**Table 3:** Proteolytic activity of VpSP37.

Specific substrates	VpSP37 activity
BOC-Gln-Ala-Arg-AMC	52,03 ± 0.6 μmol/min
BOC-Ala-Ala-Ala-AMC	n.d.
Suc-Ala-Ala-Pro-Phe-AMC	n.d.

The protease activity using different substrates was measured at concentration of 0.025 mM for each substrates. The change of fluorescence for min was converted to micromoles liberating 7-amino-4-methilcocumarin (AMC) per minute via a standard curve with a known amount of AMC.

VpSP37 efficiently cleaved the trypsin substrate BOC-Gln-Ala-Arg-AMC; conversely no cleavage was observed for the elastase specific peptide BOC-Ala-Ala-Ala-AMC or chymotripsin specific peptide Suc-Ala-Ala-Pro-Phe-AMC. Moreover to further determine the substrate specificity and verify that VpSP37 is inhibited by serine protease inhibitor, we preincubated VpSP37 with several inhibitors, and subsequently we added the purified protein to BOC-Gln-Ala-Arg-AMC or BOC-Ala-Ala-Ala-AMC peptides (Table 4). Inhibition of purified VpSP37 activity was observed in presence of serine protease inhibitors (Aprotinin and Leupeptin) whereas VpSP37 was not affected by the presence of the metal chelator, EDTA.

**Table 4:** Effect of protease inhibitors on VpSP37.

Inhibitor	Final concentration (μM)	TLA Inhibition (%)
Aprotinin	1	98,2±1
EDTA	10	n.d.
Leupeptin	50	96,5±2

Those results indicate that the purified VpSP37 belongs to serine proteases family with trypsin-like activity and does not require divalent metal ions to be active.

**Fig 7:**
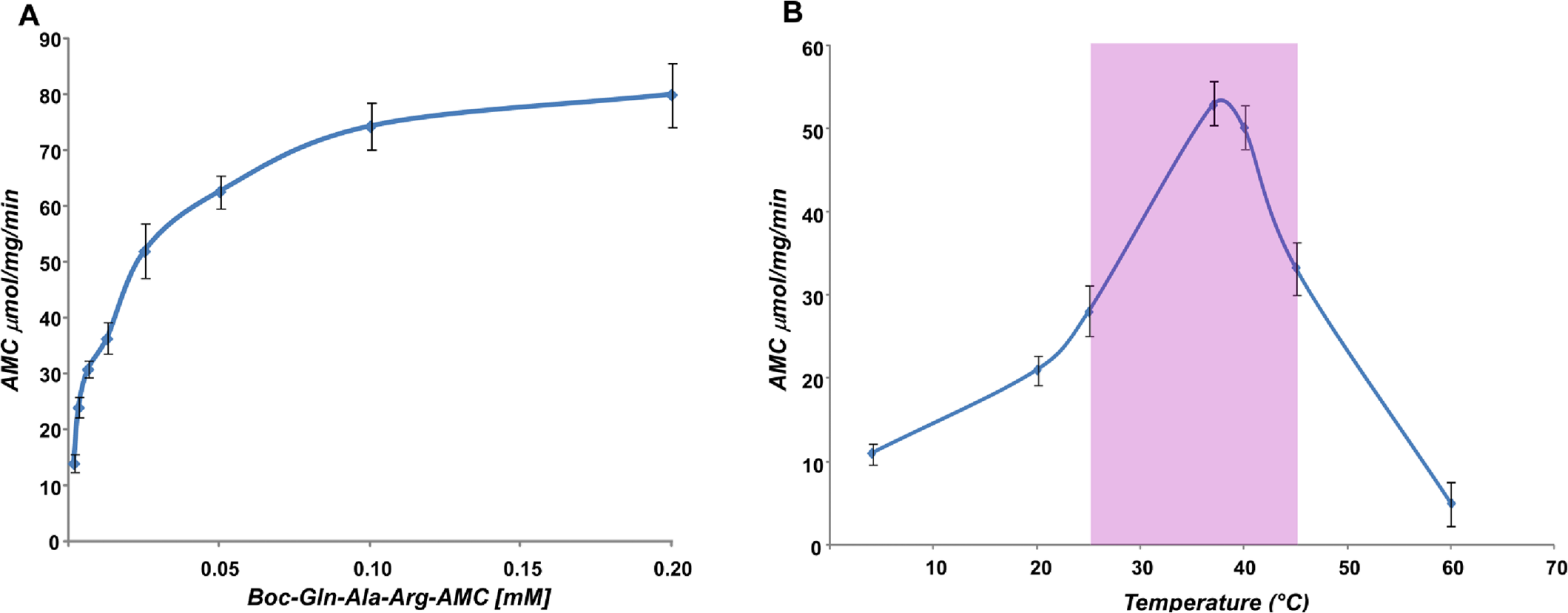
Protease activity of purified enzyme. (A) Representative plot V/[S] of VpSP37, a single experiment is showed. The protease activity was measured at different concentration of BOC-Gln-Ala-Arg-AMC to calculate *Km* e *Vmax* via non linear regression model using data from several experiments performed in triplicate. The change of fluorescence for min was converted to micromoles liberating 7-amino-4-methilcocumarin (AMC) per minute via a standard curve with a known amount of AMC. (B) Evaluation of TLA in response to temperature. The protease activity was measured at different temperatures with equal amount of BOC-Gln-Ala-Arg-AMC. Fluorescence was measured for 30 min at 355nm for excitation and 460nm for emission. The activity is expressed in micromoles/mg/minute.

To better characterise the protease activity, kinetic parameters such as *V*_*max*_ and *K*_*m*_ were calculated using different concentration of BOC-Gln-Ala-Arg-AMC (Fig 7A). Measurement of these parameters retrieved *V*_*max*_ and *K*_*m*_ values of 78.6 ± 0.8 μmol/min and 0,025 mM ± 0.0020 respectively. Moreover, as shown in Fig 7B, we tested the serine protease activity at temperatures ranging from 4°C to 60°C. Although the major activity was found in a range comprised between 35°C and 40°C, the enzyme showed half of maximal activity at 25°C. Since tissue dissociation procedures require temperature lower than 37°C to provide highly viable cells for for tranplantation [46], it is reasonable to adopt the use of this protease in procedures of tissue dissociation.

### Acceleration of tissue dissociation byVpSP37

The mechanism through which proteases break the extracellular matrix (ECM) is poorly understood. Crude extracts from the gram-positive bacteria *Clostridium histolyticum*, which have been mainly used for ECM degradation, are complex mixtures consisting of different collagenases, neutral protease, and various other enzymes including Clostripain, that possess trypsin like activity (TLA) [47]. Therefore, in addition to collagenases, proteases, play a crucial role in the islet isolation accelerating tissue dissociation. Enzymes from different sources, as Neutral protease from *C*. *histolyticum* or Thermolisin from *Bacillus thermoproteolyticus rocco*, exert a critical role in the pancreatic tissue dissociation for the release of islets. However, these enzymes have been also proven to activate pancreatic proenzymes [48] and their massive use could result in a decrease of islets yield through fragmentation and disintegration [49]. Therefore a balanced blend of enzymes are generally required in cell isolation [50–53]

**Fig 8:**
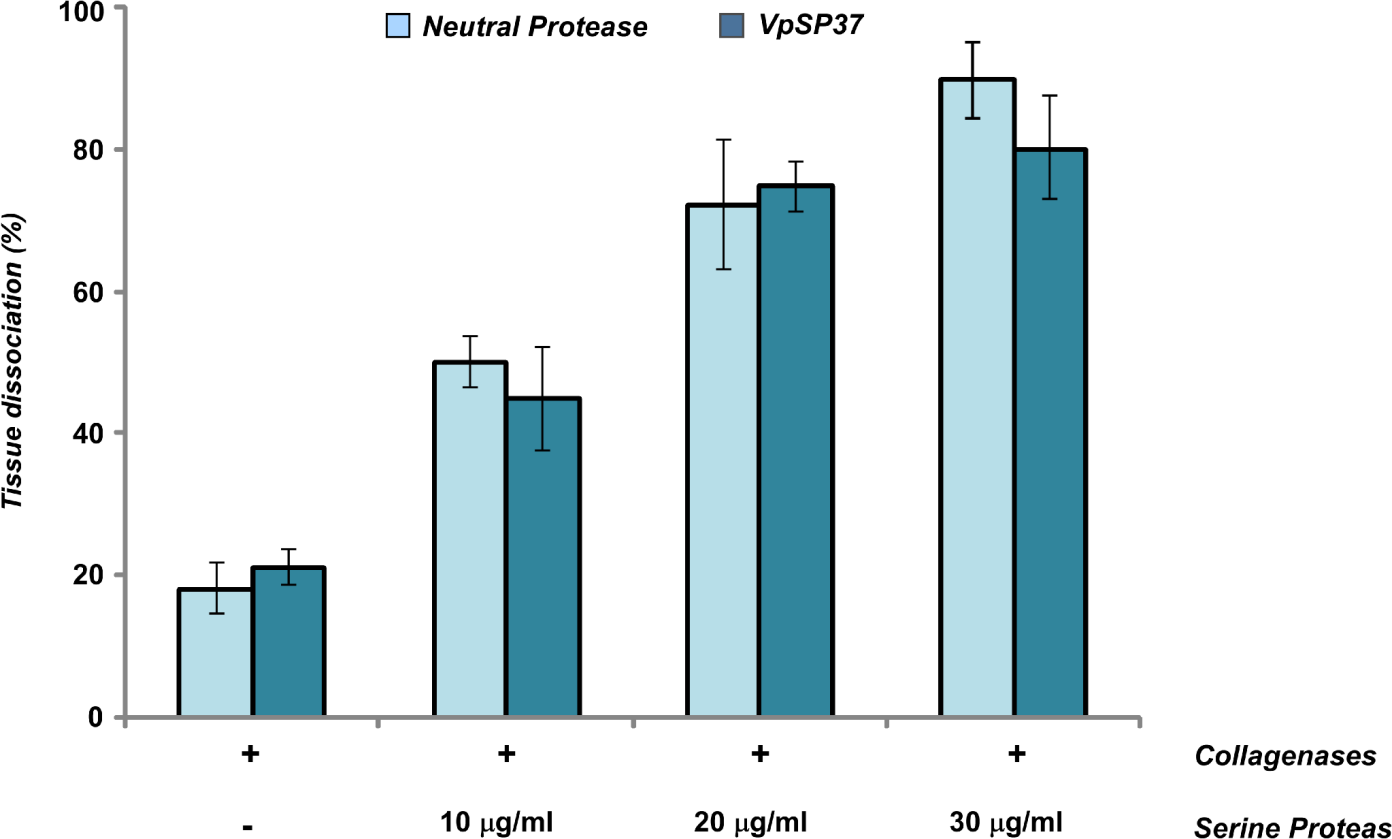
*Ex vivo* dissociation assay. Recombinant Collagenases G and H mix were used alone or in the presence of increased quantity of VpSP37 or Neutral Protease. The results are represented as means ± S.D. (n = 3).

In order to evaluate the employment of the purified VpsP37 in tissue dissociation experiments, enzyme blends of recombinant collagenases G and H and proteases (neutral protease or VpsP37) at proper ratio, as reported elsewhere [54, 55] were prepared and used to extract islets from mice pancreas. As shown in Fig 8, the purified VpSP37 was able to accelerate pancreas dissociation if compared with % of dissociation resulting from the exclusive use of collagenases. Additionally this result is consistent with the capability in acceleration of pancreas dissociation performed from neutral proteases. Since the tissue dissociation increased in the presence of proteases, in a dose dependent manner, these data suggest the possibility of using of the VpsP37 from *V*. *parahaemolyticus* B2 to enhance tissues dissociation.

**Fig 9:**
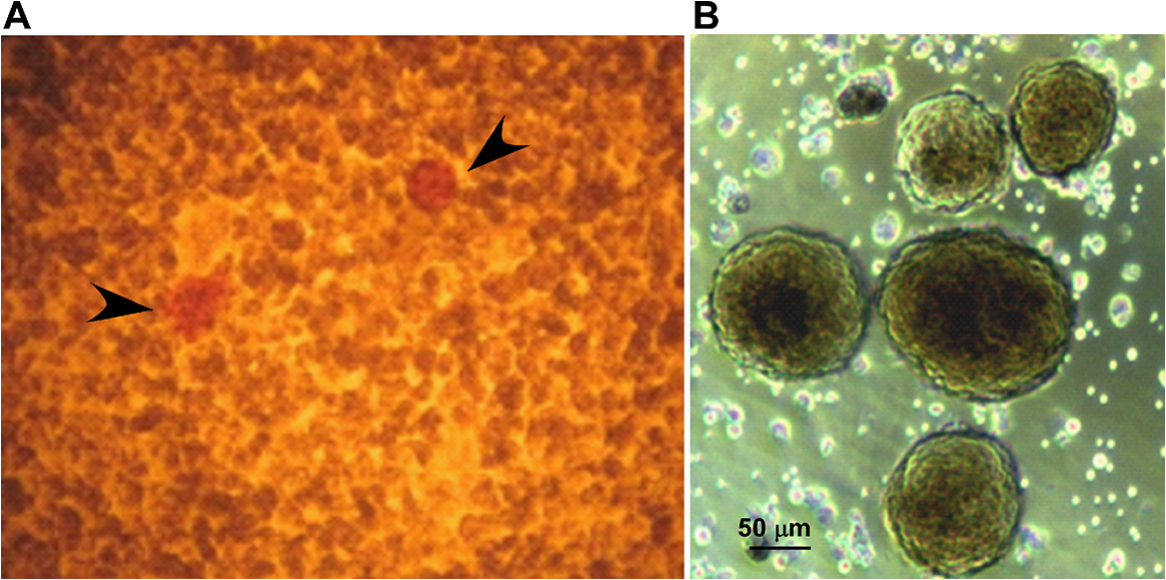
The use of VpSP37 provides viable Islets of Langerhans. (A) After pancreas disgregation, cells were stained with Dithizone to identify the Islets (marked by the arrow) as described in Materials and Methods section. (B) Islets of Langerhans were purified and cultured on selected media.

**Fig 10:**
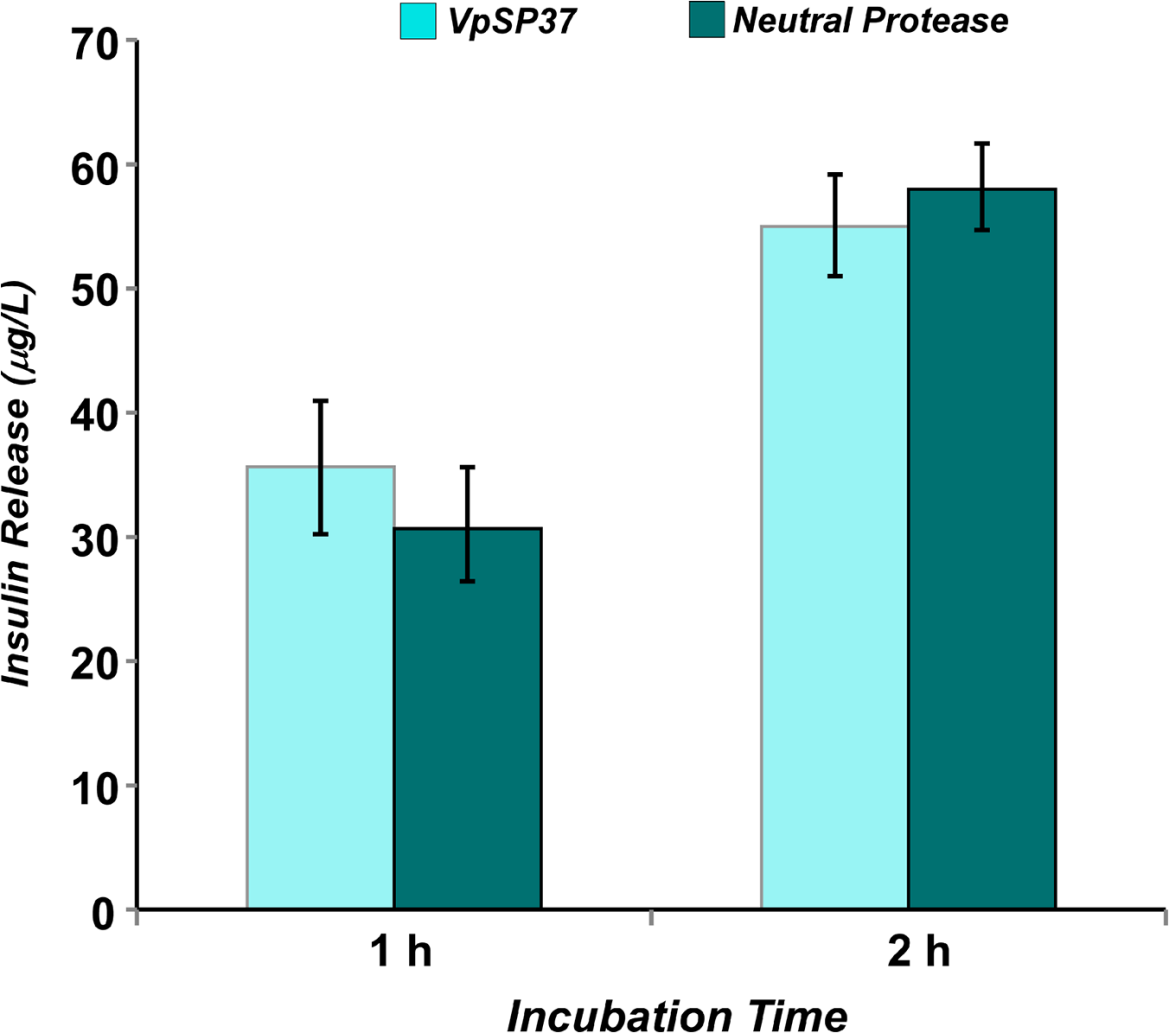
The use of VpSP37 provides functional Islets of Langerhans. The release of insulin from VpSP37 and Neutral Protease purified Islets was measured after 1h and 2h of incubation. Equal amount of Islets (10 per well) was used. The results are represented as means ± S.D. (n = 3).

After tissue dissociation, an aliquot of cells were stained with Dithizone, which specifically binds to zinc present in ß cell insulin granules and viable Islets of Langerhans were observed (Fig 9A). The purified Islets showed a morphology in which cells forming the Islets are able to maintain the required cell-cell contacts. Additionally, Islets were cultured and insulin production has been analyzed. As reported in Fig 10, the purified VpSP37 was able to provide Islets producing insulin similarly to those resulted from the use of Neutral Protease. Furthermore, insulin production do not decrease during the period of culture. Therefore the employment of VpsP37 in tissue dissociation experiments provides functionally viable, dissociated cells.

## Materials and Methods

Housing and husbandry of animals were carried out in accordance to the best practices developed in order to optimize animal health. However, all facilities and procedures complied with the Directive 2010/63/EU and the ARRIVE guidelines. The experiments were carried out in the laboratory of the STEBICEF department at University of Palermo, such structure was authorized to perform animal experimentations with the decree of the Ministry of Health 258/95. Experiments have been carried out during the 2013, in compliance with local laws, in particular the communication to professor Giulio Ghersi of research project, approved in 2013, “Enzimi per la purificazione di cellule da utilizzare in medicina rigenerativa e nella ingegneria tissutale” within the meaning of the Ministry of Health legislative decree 116/92. At the time the ethics committee was represented by this Ministry which approved the study and did not suggest any modification to project. Mice were anesthetized with isoflurane and sacrificed by cervical dislocation.

### Isolation of bacterial strain

A sterile cotton stick was introduced in the mouth of moray eel *M*. *helena*, then streaked directly on Luria Bertani (LB) Agar plate (DIFCO). After 1 day incubation at 30°C, hundreds of transparent pale yellow and flat colonies, all very similar to each other were observed on the plate. Colonies were streaked to purity and cells from few identical colonies were Gram stained and examined under light microscope.

The whole 16S gene was amplified by PCR colony using the universal bacterial 27f-CM/1492r primer set as described elsewhere [56, 57] in a 30 l volume reaction containing 1 l of the lysed colony, 0.2 M of each primer, 0.2 mM of dNTPs and 0.75 Units of One *Taq* DNA Polymerase (NEW ENGLAND Biolabs, Ipswich, MA.). The PCR was carried out under the following conditions: 94°C for 30 sec, 30 cycles of 30 sec at 94°C, 1 min at 50°C and 1.5 min at 68°C; followed by a final extension at 68°C for 5 min. The PCR product was visualized on ethidium bromide-stained 1% agarose gel. The amplicon was purified using a NucleoSpin Gel and PCR Clean-up (Macherey-Nagel, Düren, Germany) and commercially sequenced. The sequence was subjected to the Ribosomal Database Project (RDP) classifier software analysis (http://rdp.cme.msu.edu/classifier/classifier.jsp), and to BLAST search (http://www.ncbi.nlm.nih.gov/blast/) The 16S rRNA gene sequence of strain B2 has been submitted to the DDBJ /EMBL/ Genbank database under the accession number KP452506.

### Purification and characterization of serine proteases

Single colonies of *V*. *parahaemolyticus* were inoculated in LB medium (Fisher) and grown for 16 h at 30°C.

Exhausted culture broth (1.5 litre) was centrifuged at 6000 r.p.m to eliminate cells. Then it was subjected to fractionation with 60% ammonium sulphate. The precipitated proteins were suspended in Tris-HCl 20 mM pH 7.5, 0,025 mM NaCl, and dialyzed for 24 h at 4°C against repeated changes in the same buffer (after 8 and 16 h). After dialyses, proteins were quantified and used for enzymatic activity and for SDS-PAGE analyses. For fractionation, Ionic Exchange Chromatography (Q sepharose High Performance, GE Healthcare Life Sciences, Uppsala, Sweden) was performed using AKTA Start chromatographyc system (GE Healthcare Life Science, Uppsala, Sweden) as follow: total protein was loaded in 5 ml column. After washing with Tris-HCl 25 mM, 0,025 NaCl pH 7.4, proteins were eluted with NaCl gradient (0,025–0,5 mM). The positive fractions were pooled and observed in SDS PAGE and zimography. Fraction enriched in trypsin like activity was separated using HI Prep 16/60 Sephaclyl S-200 HR (GE Healthcare Life Sciences, Uppsala, Sweden). The sequence of purified protease was determined by automated Edman degradation using a Perkin Elmer protein sequencer.

### SDS elettroforeses and Zymography

Sodium dodecyl sulphate-polyacrylamide gel electrophoresis (SDS-PAGE) was carried out as described by [58], after electrophoresis, the gels were stained with 0.25% Coomassie Brilliant Blue G-250. The molecular weight of the enzyme was estimated using a molecular weight markers. Zymography was performed on native-PAGE [53]. After electrophoresis, gelatin zymographies were incubated for 24 hours at 37°C in two developing buffers: Activator buffer containing 2 mmol/L CaCl_2_;, Tris-HCl buffer (50 mmol/L; pH 7.4), containing 1.5% Triton X-100 and 0.02% Na Azide plus inhibitor buffer Tris-HCl buffer (50 mmol/L; pH 7.4), containing 1.5% Triton X-100 and 0.02% Na Azide plus 2 mmol/L EDTA to inhibit any gelatinase activity. After incubation, gel were stained using Coomassie Brilliant Blue G-250.

### Sequence and Structural Analyses

The deduced amino acid sequences of VpSP37 were compared to other known sequences, using the BLAST algorithm at NCBI.

Signal peptides, functional sites and domains in the predicted amino acid sequences were predicted by the Simple Modular Architecture Research Tool (SMART) program (http://smart.embl-heidelberg.de/), the InterPro database (http://www.ebi.ac.uk/interpro/), the Pfam database (http://pfam.sanger.ac.uk/), the PROSITE program (http://prosite.expasy.org/), SignalP 4.1 Server (http://www.cbs.dtu.dk/services/SignalP/) and the Eukaryotic Linear Motif resource (ELM) for Functional Sites in Proteins (http://elm.eu.org/elms/browse_elms.html). Multiple sequence alignments were performed using the T-Coffee program at the European Bioinformatics Institute (http://www.ebi.ac.uk/Tools/msa/tcoffee/) and rendered using the ESPript 3.0 server (http://espript.ibcp.fr/ESPript/ESPript/).

VpSP37 3D structures were reconstructed by homology modelling via the Protein Homology/analogy Recognition Engine 2.0 (Phyre 2) software [59], (http://www.sbg.bio.ic.ac.uk/phyre2/html/page.cgi?id=index), using the intensive modelling mode. Candidate structures for homology modelling were selected according to pair wise alignment and cysteine distribution. The 3D structures of six different templates (4lk4A_; 4durA_; 2b9lA_; 2f83A_; 4hzhB_; 3nxpA) were used as template. Validation of the structural protein models was performed by assessing the Ramachandran plots. Cycles of clash minimisation were also performed for the refinement of structures.

### Substrate specific activity determination and inhibition

To investigate the proteases and collagenolytic activities, we measured them using a modification of the collagen digestion method [60, 61] in which the enzymes were incubated for 5 hours with native bovine Achilles tendon collagen or casein (Sigma-Aldrich, St. Louis, MO, USA) at 37°C. The collagen and casein digestion were determined using the colorimetric ninhydrin process [61]. The amino acids released are expressed as micromoles leucine per milligram dry weight of enzyme. One unit equals one micromole of leucine equivalents released from collagen in 5 h at 37°C, pH 7.5, under the specified conditions.

BOC-Gln-Ala-Arg-AMC, BOC-Ala-Ala-Ala-AMC and Suc-Ala-Ala-Pro-Phe-AMC (PeptaNova GmbH, Keplerstr, Sandhausen, Germany) specific peptides, at concentrations reported in Table 3, were used to investigate the specific activity of VpSP37. The buffer system for analysis was a TES buffer, pH 7.4. Unless otherwise stated, 200 μL reactions were set up in microtiter wells and incubated at 37°C. Fluorescence was measured for 30 min at 355nm for excitation and 460nm for emission using the Biotek Synergy HT microplate reader (BioTek, Winooski, VT, USA). Enzyme-free reactions were used as a negative control and background fluorescence was subtracted from each value. All experiments were done in triplicate. Inhibition assays were performed using Aprotinin (Sigma-Aldrich, St. Louis, MO, USA), Leupeptin (Calbiochem, Merck KGaA, Darmstadt, Germany) and EDTA (Sigma-Aldrich, St. Louis, MO, USA) at concentrations reported in Table 4. The enzyme was pre-incubated with different concentrations of protease inhibitors at 37°C for 15–40 min. After adding BOC-Gln-Ala-Arg-AMC 0.025 mM, the residual enzyme activity was measured. The change of fluorescence for min was converted to micromoles liberated 7-amino-4-methylcocumarin (AMC) per minute via a standard curve with a known amount of AMC. Nonlinear regression model was used for determination of *Km* and *Vmax* parameters using appropriate set of experimental data.

### Enzymatic activity *in vitro* and *ex vivo*

For tissue dissociation experiments recombinant collagenases (Abiel srl, Palermo, Italy), without residual proteases activity, were used. Moreover the best concentration (1 mg/ml) and the ratio of collagenases G and H was previously reported [49]. All surgeries were performed under anesthesia, and maximal efforts were made to minimize suffering. Adult Balb/c mice were anesthetized with isoflurane (Sigma-Aldrich, St. Louis, MO, USA) and sacrificed by cervical dislocation. To evaluate the enzyme activity in *ex vivo* experiments, the pancreas was perfused with Hank’s balanced salt solution at pH 7.5 (Life Technologies, Carlsbad, CA, USA) without calcium and magnesium in order to cause distension of the pancreas [55]. Pancreas was than weighed, chopped and divided in aliquots of 250 mg for tube. Each aliquot was digested in 1 ml volume with collagenases mix at different percentage for different time of digestion at 37°C.

After digestion, the tissue was processed and filtered using 0.419 mm wire mesh. The undigested pancreas was weighed in order to measure the dissociation percentage. All experiments were conducted in triplicate. After the first experiment we perform the final experiment using VpSP37 at the optimal concentration of 30μg/mL. The digested pancreas was pooled after centrifugation and an aliquot was stained with 50 μg/mL Dithizone (DTZ, Sigma-Aldrich, St. Louis, MO, USA) to identify functional Islets. Then total digested pancreas was purified using Histopaque1077 (Sigma-Aldrich). Isolated islets were cultured in RPMI 1640 (Life Technologies, Carlsbad, CA, USA) supplemented with 10% fetal bovine serum (Life Technologies, Carlsbad, CA, USA), 1% penicillin-streptomycin (Life Technologies, Carlsbad, CA, USA), and 0.5 μg/mL fungizone (Life Technologies, Carlsbad, CA, USA) at 37°C in humid conditions with 5% CO_2_ as described elsewhere [55]. Islets were microscopically observed and the functionality was assessed evaluating the release of insulin to confirm vitality. Specifically, equal number of Islets (10/well) purified with 30μg/mL VpSP37 and Neutral Protease (SERVA Electrophoresis GmbH, Heidelberg, Germany), were pipet into a 24 well plate containing sufficient pre-warmed RPMI 1640 and insulin release was evaluated at 1h and 2h using Ultrasensitive Mouse Insulin ELISA (Mercodia, Uppsala, Sweden). The experiments were performed in triplicate.

## Conclusions

The identification of new proteases from marine environment working at temperature lower than 37°C represents an important goal for industrial biotechnology. In this work we isolated a *Vibrio* strain putatively assigned to the species *parahaemolyticus* from the oral cavity of *M*. *helena*. A synergic approach based on functional biochemical fractionation and computational analysis allowed us to purify and characterise a novel serine protease from *V*. *parahaemolyticus* strain. Although proteases related to trypsin are usually produced by eukaryotic organisms, VpSP37 showed an overall 3D structure corresponding to the typical trypsin/chymotrypsin fold, in addition to the characteristic hallmark for trypsin.

In addition to the kinetic properties of VpSP37, ex *vivo assays* were performed to evaluate the activity of this enzyme in Islet of Langherans isolation from the mouse’s pancreas. Maximizing the yield of viable, functionally dissociated cells, represents the main challenge in the tissue dissociation experiment. Indeed, procedures for cell isolation from dissociated tissues imperatively require a balanced enzyme activity against different components of ECM. Specifically, the optimization of isolation techniques is a prerequisite for successful transplantation to replace β cell function. Moreover the fact that VpSP37 showed half of maximal activity at 25°C raises the possibility of its employment in islet isolation techniques. The use of VpSP37 will contribute considerably to islet isolation outcome preserving the functional activity of the purified islet since its activity was retained at temperature lower than 37°C. Thus, enzyme blend composition, time and temperature of digestion need to be standardized in order to preserve the functional activity of the purified Islet of Langherans. Experiments herein showed strongly suggested the use of VpSP37 as a component of enzyme blend for tissue dissociation procedures.
